# Extra-pulmonary tuberculosis: A retrospective study of patients in Accra, Ghana

**DOI:** 10.1371/journal.pone.0209650

**Published:** 2019-01-09

**Authors:** Sally-Ann Ohene, Mirjam I. Bakker, John Ojo, Ardon Toonstra, Doris Awudi, Paul Klatser

**Affiliations:** 1 World Health Organization Country Office, Accra, Ghana; 2 KIT Health, Royal Tropical Institute (KIT), Amsterdam, The Netherlands; 3 School of Public Health, University of Ghana, Legon, Accra, Ghana; 4 National AIDS/STI Control Program, Ghana Health Service, Accra, Ghana; 5 Department of Global Health, Academic Medical Center, Amsterdam Institute of Global Health and Development, Amsterdam, The Netherlands; Fundació Institut d’Investigació en Ciències de la Salut Germans Trias i Pujol, Universitat Autònoma de Barcelona, SPAIN

## Abstract

**Background:**

Information on extrapulmonary TB (EPTB) patients is limited in many African countries including Ghana. The study objective was to describe the epidemiology of EPTB patients diagnosed from different categories of health facilities in Accra, Ghana compared to pulmonary TB (PTB) patients and identify risk factors for mortality among EPTB patients.

**Method:**

We conducted retrospective analyses of demographic and clinical data accessed from medical records of EPTB and PTB patients from different types of health facilities from June 2010 to December 2013. Factors at diagnosis associated with EPTB compared to pulmonary TB (PTB) and factors associated with treatment outcome death among EPTB patients were assessed using logistic regression.

**Results:**

Out of 3,342 new TB patients ≥15 years diagnosed, 728 (21.8%) had EPTB with a male: female ratio of 1.17. The EPTB sites commonly affected were disseminated 32.8%, pleura 21%, spine 13%, and Central Nervous System (CNS) 11%. Treatment success rate for EPTB was 70.1% compared to 84.2% for PTB (p<0.001). In logistic regression, HIV positivity (adjusted Odds Ratio [aOR] 3.19; 95% confidence interval [CI] 2.69–3.79) and female gender (aOR 1.59; 95% CI 1.35–1.88) were found to be significantly associated with EPTB compared with PTB. Older age, being HIV positive (aOR 3.15; 95% CI 1.20–8.25) and having CNS TB (aOR 3.88; 95% CI 1.14–13.23) were associated with mortality among EPTB patients. While more EPTB patients were diagnosed in the tertiary hospital, health facility type was not associated with mortality.

**Conclusion:**

EPTB patients in Accra have a worse treatment outcome compared to PTB patients with mortality of EPTB being associated with HIV, older age and CNS TB. Being HIV positive and female gender were found to be significantly associated with EPTB. Increased awareness of these factors may facilitate early case finding and better management outcomes for these patients.

## Introduction

Despite major strides in prevention, diagnosis and treatment, tuberculosis continues to be a major leading cause of death globally [1]. An estimated 1.67 million people died from TB in 2016 [1]. The causative organism *Mycobacterium tuberculosis*, which is predominantly air-borne, affects the lung causing pulmonary TB. When TB is bacteriologically confirmed or clinically diagnosed in other parts of the body other than the lung such as the abdomen, meninges, genitourinary tract, joints, bones, lymph nodes and skin it is classified as extrapulmonary tuberculosis (EPTB). The prevalence of EPTB among new and relapse TB cases globally in 2016 was 15% [1]. The lowest prevalence (8%) was recorded in the WHO Western Pacific Region while the highest (24%) was recorded in the Eastern Mediterranean. The figure for the African Region was 16% [1].

The African Region, which has 13% of the world’s population, accounted for 23% of the 918,011 EPTB cases reported globally in 2016 [1]. There is variation in the proportion of EPTB among TB cases in Africa with 5.2%, 31.7% and 41.3% reported for Nigeria and Ethiopia and Djibouti respectively [2]. EPTB rates are even higher in the North African countries with Morocco and Algeria reporting 44.4% and 60% respectively [2]. Ghana, a high HIV/TB burden country, with an estimated 28 million population reported 14,675 TB cases in 2016 [3]. The proportion of EPTB patients reported among new TB cases has been in the range of 8 to 10% over the period between 2006 and 2016 [3,4]. Even though data from the Ghana National TB Control Program (NTP) indicate that the prevalence of EPTB among TB patients has remained fairly stable there is concern about the mortality among EPTB patients which almost doubled from 7.8% in 2006 to 14% in 2012 [4,5]. The investigation of contributory factors to this mortality rate was highlighted as an area of interest for research in the NTP 2015–2020 Strategic Plan [5].

Various risk factors reported to be associated with EPTB include immunosuppression, HIV infection, male gender and younger age [6–13]. On the other hand, other studies have found females and increasing age to more associated with EPTB [14–17]. The sites of EPTB vary by age group and gender across different populations studied, however lymph nodes and pleura invariably feature among the top reported sites from a myriad of studies [11,13,15,16,18,19]. In view of the peculiarities of EPTB including the atypical nature of presentation, understanding the epidemiology of EPTB in diverse settings is of interest given the relevance of EPTB to TB control [14]. Except for two teaching hospital setting studies on EPTB conducted in Ghana, studies on the EPTB in-country are limited [20,21]. The current study therefore sought to highlight the epidemiology of EPTB patients diagnosed from different categories of health facilities in Accra compared to pulmonary TB (PTB) patients and identify risk factors for mortality among EPTB patients.

## Methods

The study was a retrospective secondary data analyses making use of the database of TB patients diagnosed from June 2010 to December 2013 during a TB case finding initiative implemented in 11 health facilities in Accra, the capital of Ghana. The details of the TB finding initiative were described elsewhere [22]. Accra, with a population of 1.7 million in the 2010 census, recorded HIV prevalence of 2.1% among antenatal clinic attendees over the period of 2010 to 2013 [23,24]. The facilities from which the study participants were derived included outpatient departments (OPD), HIV clinics and diabetes clinics in polyclinics, general hospitals, a regional hospital and a teaching hospital. The participants from the teaching hospital were from the HIV clinic only and did not include patients from the OPD or other clinics. These facilities accounted for 70% of TB cases in Accra at the time. From the TB case finding initiative database which consisted of 3,704 records, the participants for this study were selected using the following inclusion criteria: patients 15 years and older newly diagnosed with smear positive pulmonary TB, smear negative pulmonary TB or extra-pulmonary TB. The exclusion criteria were patients less than 15 years and those previously treated for TB. The classification of PTB and EPTB by the National Tuberculosis Control Program (NTP) in Ghana falls in line with WHO guidelines [25]. With the exception of cerebro-spinal fluid (CSF) samples, which were usually quite small in volume, EPTB samples for microscopy and culture were taken through a decontamination process to get rid of other bacteria using a 4% sodium hydroxide (NaOh) and N-acetyl L-cysteine (NALC) preparation. [26] An equal volume of NaOH-NALC solution was added to the sample for a quarter of an hour followed by neutralization with phosphate buffer solution pH 6.8. The preparation was then subjected to centrifugation for concentration of the specimen and to wash off the sodium hydroxide reagent. The decontaminated concentrated sample was then inoculated for culture and smear preparation. At the time of the case finding initiative, sputum smear samples for examination under light or light emitting diode (LED) microscopy were processed using Ziehl Nielsen staining method. Sputum smear positive PTB was defined as a patient with acid fast bacilli in at least one sample of sputum. A patient was considered to have sputum smear negative PTB if he or she had two sputum smears negative for mycobacteria on microscopy, but Chest X-ray showed evidence consistent with active tuberculosis. EPTB was classified as per organs or systems affected exclusive of the lungs, such as lymphatic comprising of TB in lymph nodes, pleura, spine, TB in bones and joints other than the spine, central nervous systems CNS (TB meningitis, brain), abdominal and other such as genito-urinary tract. EPTB diagnosis was based on having one culture-positive specimen using fine needle aspiration biopsy or organ fluid samples such as ascetic or pleural fluid depending on the suspected site involved, or histological evidence or strong clinical confirmation of active EPTB for which the clinician makes the decision to treat with a full course of TB drugs. Culture methods available included solid culture using Lowenstein-Jensen media and liquid culture by means of Bactec Mycobacteria Growth Indicator Tube (MGIT960, BD, Sparks, USA). These diagnostic methods were available at the teaching hospital laboratory which also performed culture on samples that were delivered from other lower level facilities. In the event that a patient has EPTB in several organs, the patient is classified according to the site that is most severely affected. In NTP registration, patients diagnosed with both PTB and EPTB were registered as pulmonary TB. It is therefore not possible to distinguish which patients had both types of TB. At the time of the case-finding initiative, the same standardized first line TB drugs were used to treat new cases of EPTB and PTB for the duration of 6 months [25]. Classification of treatment outcomes were cure, treatment completed, default, died, transfer out and treatment failure as per WHO guidelines. The combination of those recorded as having been cured and completed treatment were designated as having a favorable treatment outcome. Data of study participants obtained from their medical records included age, gender, HIV status, type of TB, site of the EPTB, facility of diagnosis, year of diagnosis and treatment outcome.

Data analysis was done by means of STATA version 12. The frequencies and percentages of the respective types of EPTB in totality and then stratified by gender and the age distribution were assessed. Multi-variate logistic regression analysis was conducted to identify factors associated with EPTB relative to PTB and risk factors associated with mortality among EPTB patients. Baseline characteristics including age group, gender, HIV status were included in the first model. In the second model those with who died during treatment were compared to those successfully finishing their treatment. Age group, gender, HIV status, affected site and facility of diagnosis were all included in the second model. Adjusted OR, 95% CI and p-values were calculated for each potential predictor variable with p-value of <0.05 set as the level of significance.

Data collected for the analysis did not have any personal identifying information and was handled with strict confidentiality. Ethics approval for the study was obtained from the Ghana Health Service Ethical Review Committee.

## Results

Out of 3,704 TB patients recorded in the TB case finding initiative database, 219 children less than 15 years and 143 patients who were not new TB cases were excluded from the analysis. The study participants consisted of 3,342 new TB patients who were aged 15 years and above. The overall male female ratio was 1.68. A total of 1,443 (42.9%) of these TB patients, were from the polyclinics; 775 (23.2%) were from the general hospital; 775 (23.2%) were from the HIV clinic of the teaching hospital and 359 patients (10.7%) were from the regional hospital. There were 728 patients (21.8%) who had extra-pulmonary TB while 2,614 patients had pulmonary TB. Out of the 728 EPTB patients, 400 (55%) were diagnosed from the HIV clinic of the teaching hospital. Almost half (48.5%) of the 2,614 pulmonary TB patients, were diagnosed in the general hospitals.

**Fig 1:**
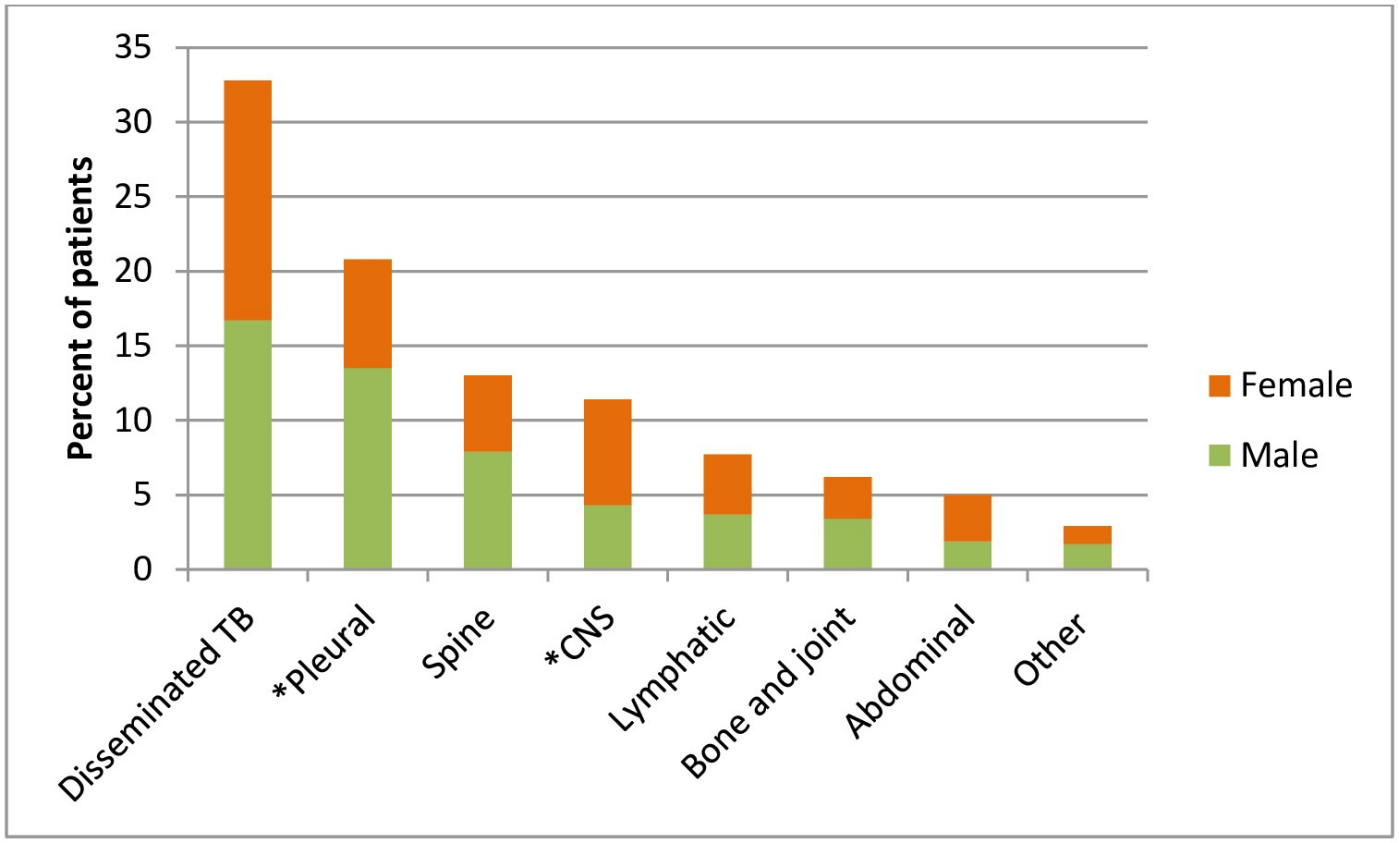
Distribution of 646 extrapulmonary patients by infection site and gender in Accra, 2010–2013. The figure shows the distribution of EPTB across various sites for males and females. A significantly higher proportion of males had pleural TB while a significantly higher proportion of females had CNS TB compared to males.

For 646 (88.7%) of the EPTB patients, the site affected was recorded. Two hundred and twelve (32.8%) of the EPTB patients had the classification of disseminated TB, while for 134 patients (18.4%) the site affected was the pleura (Fig 1). Twenty patients (2.9% of EPTB cases) making up the category of “other” had various sites affected including pericardial, genitourinary, skin, and breast. Significantly more males 25.4% (87/343) reported pleural TB compared to females: 15.6% (47/302) p<0.01, while CNS TB was more common among females 15.2% (46/302) than men: 8.2% (28/343) p<0.01. For the other categories of EPTB, almost similar proportions of males and females were affected.

Treatment outcome was documented for 665 (91.3%) EPTB patients. The overall treatment success rate was 70.1% for the EPTB patients (Table 1) with 6 patients reported to be cured. Treatment success ranged from 57.9% to 74.6% with 2012 being the year that treatment success rate was highest. The lowest treatment success rate was documented for 2013, possibly related to the low proportion of EPTB patients for which the treatment outcome was known (68%). Among EPTB patients, the overall mortality rate was 28.7%. The mortality rate was highest among those with CNS EPTB (52%) and disseminated EPTB (47%). Death rate among those with pericardial EPTB was also very high, but the number was very small (3/5 patients). Among PTB patients, the overall treatment success rate was 84.2% while the mortality rate was 12.6%.

**Table 1:** Treatment outcome of 665 EPTB patients with documented treatment outcomes out of 728 diagnosed EPTB patients in Accra from 2010 to 2013.

Year of diagnosis	Total number of EPTB patients diagnosed (% with treatment outcome documented)	Cured n (%)	Treatment completed n (%)	Treatment failed n (%)	Died n (%)	Lost to follow up n (%)	Not evaluated n (%)	Treatment success n (%)
**2010**	175 (98.9)	5 (2.9)	116 (67.1)	0	49 (28.3)	3 (1.7)	0	121 (69.9)
**2011**	215 (98.6)	0	154 (72.6)	0	57 (26.9)	0	1 (0.5)	154 (72.6)
**2012**	182 (95.1)	1 (0.6)	128 (74.0)	0	43 (24.9)	1 (0.6)	0	129 (74.6)
**2013**	156 (68.6)	0	62 (58.0)	0	42 (39.3)	0	3 (2.8)	62 (57.9)
**Total**	728	6 (0.9)	460 (69.2)	0	191 (28.7)	4 (0.6)	4 (0.6)	466 (70.1)

**Fig 2:**
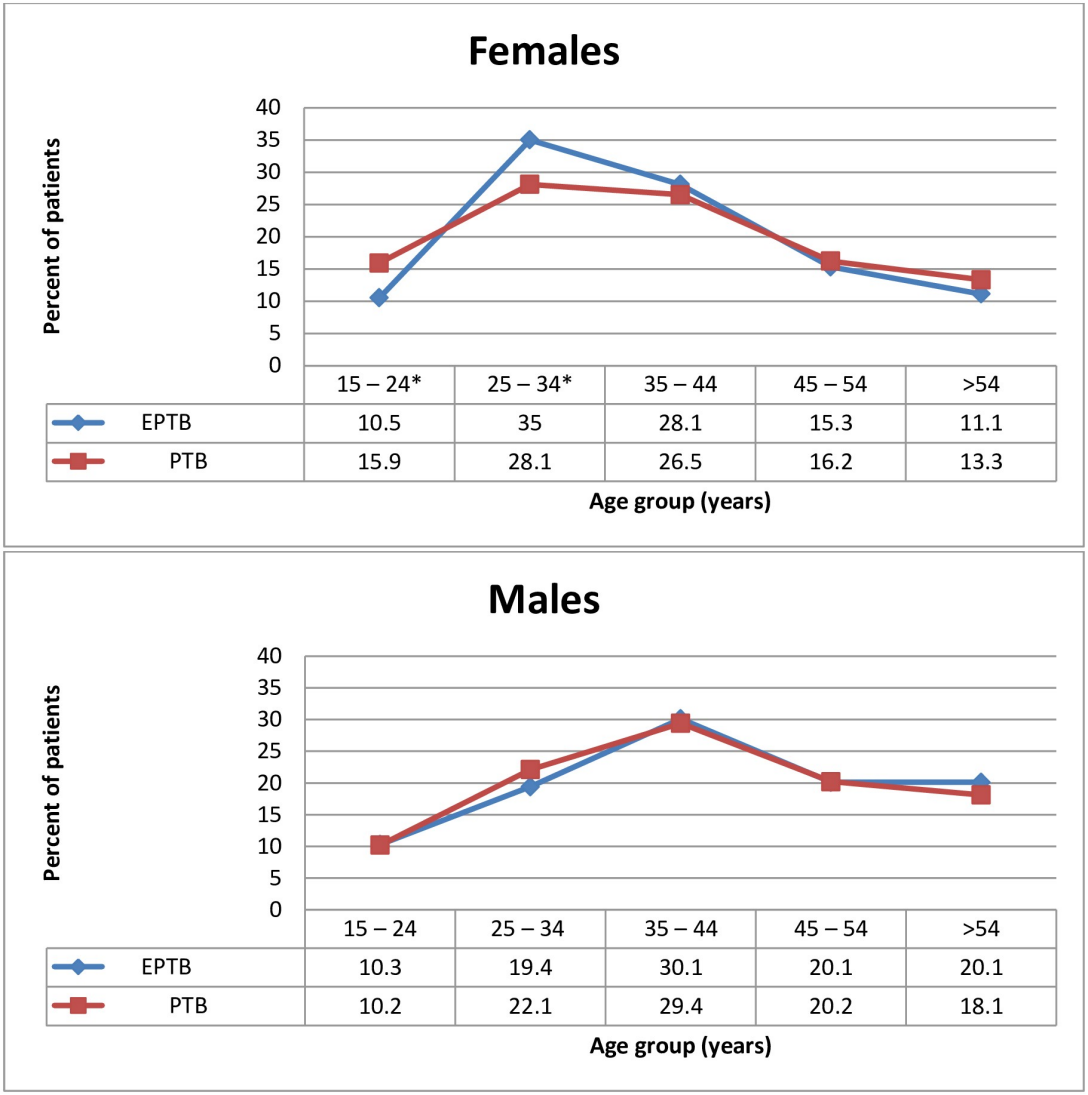
Age distribution of PTB and EPTB by gender among TB patients in Accra, 2010 to 2013. PTB: pulmonary TB EPTB: extrapulmonary TB.

There was male predominance for both EPTB and PTB. For EPTB, the male: female ratio was 1.17:1 while for PTB, the male: female ratio was 1.87:1. The proportion of females with EPTB was significantly more than the proportion of females with PTB (p < 0.001) Table 2. The age distribution for both EPTB and PTB by gender is shown in Fig 2. Treatment success was significantly higher among PTB patients than EPTB patients.

**Table 2:** Comparison of EPTB and PTB across demographic and clinical variables among TB patients in Accra 2010 to 2013.

Characteristic	EPTB n (%)	PTB n (%)	p-value
**Gender**			
Male	392 (54.0)	1,698 (65.1)	
Female	334 (46.0)	909 (34.9)	<0.0001
**Age (years)**			
Median	38	39	
Mean	40.4 (13.5)	40.6 (14.3)	
15–34	270 (37.1)	950 (36.4)	0.722
35–54	342 (47.0)	1232 (47.2)	0.928
≥55	116 (15.9)	430 (16.5)	0.601
**HIV status**			
Positive	454 (62.4)	895 (32.4)	
Negative	268 (36.8)	1,686 (64.5)	<0.0001
**Facility**			
Polyclinic	72 (9.9)	703 (26.9)	<0.002
General Hospital	165 (22.7)	1,268 (48.5)	<0.0001
Regional Hospital	91 (12.5)	268 (10.2)	0.5411
Teaching Hospital	400 (55.0)	375 (14.3)	<0.0001
**Treatment Outcome**			
Treatment success	466 (70.1)	2118 (84.2)	<0.0001

The HIV status was known for almost all the TB patients (98.8%). Among these patients for whom the HIV status was known, about two fifths (40.8%) were HIV positive. About 62% of EPTB patients were HIV positive compared to 32.4% of the PTB patients. The participants in the study from the teaching hospital came from the HIV clinic and as such all these TB patients were HIV positive. Among these HIV positive patients from the teaching hospital, 52% had EPTB. Among those who were HIV positive in the regional hospital, 20.5% had EPTB while the general hospitals and polyclinics had 8.3% and 6.3% respectively of the HIV positive patients having EPTB. The cure rate and treatment success rate for HIV positive EPTB was 1.3% and 51.8% respectively while the figures for HIV positive PTB patients were 26.8% and 66.7% respectively.

The multivariate logistic regression analysis showed that EPTB patients were more often female and HIV positive compared to PTB patients (Table 3). The likelihood of developing EPTB was more than 3 times among those with HIV. On the other hand, the age groups did not show any differential association with EPTB.

**Table 3:** Multi-variate logistic regression model determining the risk factors for developing extra pulmonary tuberculosis relative to pulmonary tuberculosis among 3,342 TB patients in Accra, 2010 to 2013.

Variable	Adjusted odds ratio	95% CI	p-Value
**Age (Years)**			
15–34	1		
35–54	0.98	0.81–1.17	0.834
≥55	0.95	0.74–1.21	0.723
**Gender**			
Male	1		
Female	1.59	1.35–1.88	<0.0001
**HIV status**			
Negative	1		
Positive	3.19	2.69–3.79	<0.0001

Table 4 shows the results of a multivariate logistic regression with mortality as outcome compared to those treated successfully among EPTB patients. Risk factors for death included increasing age, HIV positivity and CNS TB. Going by the cut off for statistical significance the type of health facility attended by the EPTB patients did not appear to be associated with mortality, however mortality among EPTB patients attending the teaching hospital was worse than for the other facilities with the p value just falling shy of the level of significance at 0.077.

**Table 4:** Association of clinical factors and facility type with mortality among 657 EPTB patients in Accra 2010 to 2013.

Variable	N	% Died	Adjusted odds ratio	95% CI	p-Value
**Age (Years)**					
15–24	68	8.8%	1		
25–34	176	33.5%	3.79	1.32–10.84	0.013
35–44	191	31.4%	2.54	0.88–7.23	0.082
45–54	115	31.3%	3.57	1.19–10.68	0.023
>54	107	28.0%	5.53	1.77–17.19	0.003
**Gender**					
Female	301	32.6%	1		
Male	354	26.0%	0.96	0.63–1.47	0.850
**HIV status**					
HIV-	249	8.8%	1		
HIV+	402	41.5%	3.15	1.20–8.25	0.020
unknown	4	33.3%			
**Site affected**					
Abdominal	30	13.3%	1		
Bone and joint	37	5.4%	0.56	0.14–3.64	0.547
CNS	67	53.7%	3.88	1.14–13.23	0.030
Disseminated	188	47.3%	2.85	0.89–9.11	0.078
Lymph nodes	47	14.9%	1.04	0.26–4.25	0.952
Other	13	15.4%	0.98	0.14–6.65	0.982
Pericardial	5	60.0%	4.15	0.49–35.30	0.193
Pleural	118	16.1%	0.84	0.24–2.89	0.780
Spine	78	12.8%	1.11	0.28–4.34	0.880
**Facility**					
General Hospital	148	11.5%	1		
Teaching Hospital	353	43.6%	2.40	0.91–6.31	0.077
Polyclinic	69	8.7%	1.54	0.45–5.23	0.489
Regional Hospital	87	16.1%	2.15	0.82–5.56	0.116

## Discussion

This study elaborates on EPTB diagnosed among patients attending different types of facilities and HIV clinics in Accra over a period of three and a half years and compares demographic and clinical characteristics of these patients with those having pulmonary TB. One fifth of the newly diagnosed tuberculosis had EPTB. The most common form of EPTB was disseminated TB followed by pleural TB. A favorable treatment outcome was observed for seven out of ten EPTB patients with documented treatment outcome. Being HIV positive and female gender were found to be significantly associated with EPTB compared with PTB while older age, being HIV positive and having CNS TB was associated with mortality among EPTB patients.

The reported proportion of EPTB among TB patients in our study (21.8%) fell within the range of what has been reported for other countries such as Swaziland (18.4%), Cameroon (19.4%) and Botswana (25%), which like Ghana are also classified as having a high HIV/TB burden [1,4]. In this same category of HIV/TB burden countries, Uganda and Malawi have EPTB prevalence reported of 11% and 41%, respectively, highlighting the wide variation of the EPTB prevalence across countries and the uncertainty of reasons for this observation [4,13]. Interestingly, the prevalence in our study was relatively higher than the countrywide data in the range of 8–10% reported for Ghana [4]. It is worthy to note that our study population was derived from facilities in Accra and also consisted of patients from the country’s premier teaching hospital HIV clinic, which has a large clientele. Usually such tertiary facilities have more sensitive diagnostic options for identifying EPTB. Secondly being a referral hospital, it receives a range of patients from other facilities including possibly HIV positive EPTB patients who may have been referred because of difficulties in making a diagnosis. This may explain the preponderance of the EPTB patients from the teaching hospital and the observed prevalence of EPTB [12]. The HIV prevalence of 40% among our patients was also high compared to the 24% reported for Ghana because of the selection of the clinics for the case finding initiative which in the teaching hospital involved only the HIV clinic to the exclusion of the OPD and other clinics [4, 22].

Different studies report the pleura and the lymphatic system as the commonly affected extra pulmonary sites, while in our study disseminated TB topped the list followed by the pleura and other sites similar to what others have reported [12,13, 18, 27,28]. The rate of disseminated TB that we found was similar to what was reported in a study of EPTB patients seen in a referral hospital in the capital of Cameroon [28]. Disseminated TB is a severe form of EPTB usually associated with HIV infection with progression linked to the immuno-suppression and delayed treatment [29]. Given the HIV coinfection in our study population and with disseminated TB presenting with systemic symptoms, it is possible that delayed diagnosis may have contributed to this magnitude of disseminated TB [6,12, 20].

In consonance with other studies, our study found an association between female gender and EPTB [10,13,27,30]. While the reasons for this finding have not been clearly identified, it is suggested that genetic factors, gender differences in exposure to TB and the presence of other risk factors such as smoking could possibly be the linking factors [12,15]. Further studies are needed to clearly delineate the linkages. The distribution of the site affected by EPTB across gender however does not appear to follow a clear pattern as various studies show similar or slightly different gender predominance in one site or the other [11, 12,14,16]. The predominance of males among those with pleural TB found in our study has also been reported by other researchers [10,27,28]. Similar to what was observed in other studies, we found no association between EPTB and age [15,31].

The treatment success rate for EPTB in our study was comparable to what Gomes *et al*. reports for EPTB patients in Brazil but lower than what was reported by studies conducted in Benin, Ethiopia and India [10,15, 32,33]. On the other hand, it is higher than what was reported by other researchers in Nigeria [34,35]. Various systems of DOTS implementation may be contributory factors to the differences. The treatment success rate for Ghana has shown consistent improvement over the years with figures exceeding 85% over the course of the time during which data for our study was recorded [4,36]. This achievement has been attributed to various factors including social and biomedical factors including the enablers package, community involvement in treatment and the used of fixed dose combinations [36]. The EPTB patients in our study had a relatively lower treatment success rate than the national average for the country. Among those with poor outcomes, it was observed that death was responsible for an overwhelming majority. The rate of disseminated TB among our patients may be associated with the mortality observed given that in their study of mortality among Ghanaian TB patients, Nassikas *et al*. and Burton and colleagues showed disseminated TB as one of the highest risk factors for death [20,37]. The HIV co-infection predominance among EPTB patients compared to PTB and the finding of HIV and CNS TB as risk factors for death in our study corroborates findings from different studies and highlights the importance of early initiation of anti-retroviral therapy for the survival of HIV-infected EPTB patients [7, 10,29,37–39]. As pointed out by Ade *et al*. severe immune-suppression found in HIV patients with EPTB predisposes to these patients succumbing to opportunistic infection and ultimately death especially when anti-retroviral therapy is started late [10]. This re-iterates the importance of vigilance to facilitate early diagnosis of EPTB to improve treatment outcomes and minimize the risk of progression to advanced forms and death [40,41].

TB infection in people is mainly caused by *Mycobacterium tuberculosis* complex [42]. The data on the causative pathogens for EPTB was however not available to this study. Unlike for pulmonary tuberculosis, very little has been studied on the *Mycobacterium tuberculosis* complex species responsible for EPTB in Ghana. One study conducted in Accra however found *M*. *tuberculosis* to be the predominant organism (eighty-eight percent of the samples) with *M*. *africanum* being the only other organism accounting for the remainder [26]. In various studies on pulmonary TB in Ghana in which samples were analyzed, *M*. *tuberculosis* was also the major organism of *M*. *tuberculosis* complex isolated followed by *M*. *africanum* accounting for 73% to 97.6% and 2.4% to 23% of samples respectively [42–45]. *M*. *Bovis* was however isolated in fewer studies and accounted for 0.4 to 3% of the samples [42,44].

EPTB diagnosis in general poses a challenge given the problem of obtaining the appropriate specimen from suspected EPTB sites and the fewer *Mycobacterium* bacilli in EPTB samples limiting the identification of AFP using microscopy [41]. At the time of the TB case finding initiative, microscopy which has low sensitivity but high specificity, was the main stay of TB diagnosis in these lower level facilities though further tests such as cultures on samples could be accessed at the teaching hospital laboratory. Fewer cases of EPTB were diagnosed in general hospitals and clinics in our study and may reflect a lack of appropriate well-defined diagnostic algorithms as available for PTB as well as inadequate facility resources and clinical expertise [30]. Since generally more people access health care at lower level health facilities compared to tertiary hospitals, the cost benefit may be in favour of enabling these non-tertiary facilities using appropriate algorithms to freely access molecular tests and culture, which have higher sensitivity and specificity compared to microscopy [46]. This may improve timely diagnosis of EPTB especially among those living with HIV to ultimately reduce morbidity and mortality [41]. Our study shows that health facility type was not significantly associated with mortality. Further studies could explore possible factors at play including the association if any between the time to diagnosis and initiation of treatment at the respective facilities and treatment outcomes.

Our study has some limitations. It involved secondary data analyses with the data source being the TB-case finding initiative database. Consequently there could be potential errors in the data since it was not possible to verify the diagnosis of EPTB or assess whether some of the study participants were misdiagnosed as EPTB. Similarly, the clinical improvement of patients who completed treatment could not be verified. Secondly, it was also not possible to distinguish patients who had concurrent EPTB and PTB among the study population as this information was not clearly indicated. This is against the background that in reporting as per Ghana NTP guideline, patients with both EPTB and PTB are recorded as PTB. There was also the challenge of incomplete documentation. The data from the teaching hospital consisted only of patients from the HIV clinic which may have introduced an element of bias in the results showing the association between EPTB and HIV. This result should therefore be interpreted against the light of this potential bias. Finally, with the study population being derived from Accra, the study findings may preclude generalization to the rest of the country. Despite these limitations, a major strength is that, to our knowledge, this is the first study that uses data from different facility types in Ghana to elaborate on EPTB in comparison to PTB, the treatment outcomes of EPTB patients and the risk factors for mortality.

## Conclusion

This study in summary showed female gender and HIV co-infection as risk factors for EPTB, and HIV and CNS TB as risk factors for death among EPTB patients. Increased awareness of these factors, provision of and training in country-adapted diagnostic algorithms and making more sensitive diagnostic tools accessible may contribute to earlier case finding and diagnosis of EPTB patients especially at lower level health facilities for initiation of treatment and possibly better management outcomes [41,47].
